# Supplementary material for: Classifying Malignancy in Prostate Glandular Structures from Biopsy Scans with Deep Learning
Source: Cancers (Basel). 2023 Apr 17;15(8):2335. doi: 10.3390/cancers15082335 (PMC10136774; doi:10.3390/cancers15082335)
Supplement: Supplementary file 1 [file cancers-15-02335-s001.zip › cancers-2248963-supplementary.pdf]

cancers

MDPI

Classifying Malignancy in Prostate Glandular Structures from Biopsy Scans with Deep Learning—Supplemental Documentation

This supplemental document provides additional detail regarding prostate pathology deep learning (DL) studies performed as published in "Classifying Malignancy in Prostate Glandular Structures from Biopsy Scans with Deep Learning". For ease of the reader, section numbers in this document are made consistent with the formal paper whereby this ancillary information is most relevant. For this reason, not all section numbers provide additional information.

S1. Introduction

N/A.

S2. Materials and Methods Supplemental

S2.1. Deep Learning

S2.1.1. Hyper Parameters

The hyper parameters used to study the DL models are shown in Table S1. Since cosine annealing was used on learning rate (LR), a stochastic gradient descent (SGD) optimizer was used with a Nesterov momentum of 0.9. Other parameters of the cosine annealing curve are also shown. Dropout was used between the last CNN layer and between the two fully connected layers. Pooling was also performed between the CNN and FC layers. Note early termination was set unusually high such that it was long enough to stride across the cosine annealing window. The idea is to ensure that if the optimizer escapes a local minimum after shocking the learning rate, we train long enough to search for a new minimum.

Table S1. Hyper Parameters.

PARAMETER	VALUE	PARAMETER	VALUE
Optimizer	SGD	*Dropout*	0.65
Momentum	0.9	*Pooling*	Global Average
Batch Size	29 (typically)	*Early Term. Patience*	75
LR Function	Cosine	*Early Term Metric*	Min. val. Loss.
LR Cycle	60 epochs	*Tensor Size*	300 × 300 × 3
LR Warmup Cycles	5	*Sample-mix Rank*	3 (nominally)
LR High	0.02	*Augmentation*	Random Flips
LR Low	0.02×(*LR High*)		

S3. Results

We evaluated several popular deep CNN architectures prior to converging on a network for our study; the top eight are listed in Table S2. In the study, all of the networks were pretrained on the large image dataset, ImageNet, prior to training for histopathological discrimination. We find, the Visual Geometry Group (VGG) networks performed quite a bit better than ResNet and EfficientNet variants, based on F1-score. We observed several key performance criteria, but the selection was based on F1-score, as it provides a measure of both sensitivity and specificity. While the VGG architecture [1] is known to have a large number of parameters, many of them are contained in the dense or fully-connected classification layers, tail-end of the network. In our study, this tail was removed in place of our own trained classification layers. As a result, the performance versus

number of parameters is competitive with other networks, scoring better than the larger ResNets, and significantly better than the EfficientNet variants, as also shown in Table S2. The number of parameters for each of these networks are reduced from those trained on ImageNet, as the fully-connected or dense output classification layers are replaced with a much smaller classification network with two layers: a 32-node feature aggregation dense layer with RELU outputs to distill features, followed by a single binary node with sigmoid nonlinear function.

Table S2. Comparison of deep learning networks on GS3 versus GS4 classification.

	F1-Score	AUC	Parameters
ResNet-50	0.590	0.581	23.6M
ResNet-101	0.636	0.577	42.6M
EfficientNet-B0	0.596	0.607	4.1M
EfficientNet-B1	0.605	0.607	6.6M
EfficientNet-B2	0.563	0.582	7.7M
EfficientNet-B3	0.567	0.585	10.7M
VGG-16	0.690	0.670	14.7M
VGG-19	0.686	0.674	20.0M

As discussed in Section 2.2 of the formal paper, one of our challenges was to unify the size of all prostate pathology patches used in classification. Since the UM/MCC cohort was made up of labeled glands, and patches derived were random in size, numerous techniques were tried to present the data to CNN feature layers. We tested the sample-mix technique independently against conventional rescaling approaches as an ablation study (a proof of the technique by testing with and without the feature), as shown in Table S3. As shown, the sample-mix technique performed well, and importantly did not degrade performance for the CNN feature extractor. The ablation study has shown that the sample-mix technique may not provide a boost in performance, but results demonstrate that it is an effective approach and does not hurt performance when discriminating textural features in this domain (in our case, performance was slightly improved).

Table S3. Comparison of Image Patch Resizing Techniques.

	F1-Score	AUC
Tight Bounding Box + Resize	0.684	0.669
Square Bounding Box + Resize	0.640	0.640
Fixed-Sized Bounding Box	0.647	0.639
Sample-Mix	0.690	0.670

Initial results shown in Table S3 are not remarkable but did show promise that the deep learner is able to distinguish degrees of malignancy. The VGG-16 and sample-mix technique proved to be the top performer and most practical.

S3.1. Deep Network Performance

In addition to the results shown in the main paper, we additionally performed various generalization studies on the UM/MCC and PANDA Radboud datasets. The results shown here demonstrate how different the 2 datasets truly are. After pretraining a network on PANDA Radboud data, we then retrained the two networks on our UM/MCC dataset, one for the Benign versus GS3/4/5 case, and another for GS3 versus GS4 classification. Again, the data patches from our two data cohorts are quite different, despite both being derived from prostate pathology H&E stained WSIs. As mentioned in Section 2.1,

UM/MCC data is generated from individual glands, while PANDA Radboud has fixed-size (400 × 400) patches derived from sufficiently densely labeled areas. Despite these differences, we checked if the networks were generalizing well across sources. As Table S4 shows, cross-source generalization is quite poor. Since the network trained on UM/MCC data was first trained on PANDA Radboud data, the 3rd and 4th columns are a clear case of catastrophic forgetting, a common problem with machine learners [2]. The network that jointly trains on both sources and that discriminates source and Gleason pattern simultaneously as shown in the main document, significantly improves on these scores.

Table S4. Cross-source inference demonstrates poor performance.

	**Trained on PANDA Radboud**		**Trained on UM/MCC**	
	**UM/MCC Benign vs GS3/4**	**UM/MCC GS3 vs GS4**	**PANDA Radboud Benign vs GS3/4/5**	**PANDA Radboud GS3 vs GS4**
Accuracy	0.567 (0.40, 0.70)	0.305 (0.28, 0.34)	0.476 (0.39, 0.58)	0.529 (0.46, 0.70)
Sensitivity	0.607 (0.22, 0.95)	0.899 (0.63, 0.99)	0.521 (0.33, 0.84)	0.805 (0.152, 1.0)
Specificity	0.682 (0.23, 0.98)	0.064 (0.01, 0.22)	0.521 (0.334, 0.843)	0.305 (0.004, 0.93)
Precision	0.723 (0.36, 0.97)	0.273 (0.19, 0.33)	0.722 (0.465, 0.873)	0.529 (0.436, 0.77)
NPV	0.579 (0.28, 0.92)	0.621 (0.42, 0.75)	0.722 (0.465, 0.873)	0.671 (0.43, 1.0)
F_1-score	0.584 (0.35, 0.72)	0.419 (0.29, 0.50)	0.568 (0.473, 0.674)	0.600 (0.25, 0.69)
AUC	0.738 (0.64, 0.82)	0.454 (0.39, 0.50)	0.522 (0.40, 0.70)	0.692 (0.58, 0.83)

References

1. Simonyan, K.; Zisserman, A. Very Deep Convolutional Networks for Large-Scale Image Recognition. *arXiv* **2015**, arXiv:1409.1556.
2. McCloskey, M.; Cohen, N.J. Catastrophic Interference in Connectionist Networks: The Sequential Learning Problem. *Psychol. Learn. Motiv.* **1989**, *24*, 57. https://doi.org/10.1016/S0079-7421(08)60536-8.
